# Choosing awareness over fear: Risk analysis and free trade support global food security

**DOI:** 10.1016/j.gfs.2020.100445

**Published:** 2020-09

**Authors:** Julie Adamchick, Andres M. Perez

**Affiliations:** Department of Veterinary Population Medicine, College of Veterinary Medicine, University of Minnesota, USA

**Keywords:** Food animals, Risk analysis, Epidemic, SPS agreement, COVID-19, Food systems

## Abstract

Livestock production and global trade are key components to achieving food security, but are bedfellows with the risk for emergence and spread of infectious diseases. The World Trade Organization's Agreement on the Application of Sanitary and Phytosanitary Measures outlines provisions for member countries to protect animal, plant, and public health while promoting free trade. The capacity for risk analysis equips countries to increase access to export markets, improve local animal health and food safety regarding known hazards, and build the institutional capacity to respond to unexpected events. The COVID-19 pandemic has highlighted the need to detect, report, and implement effective response measures to emerging challenges on a local and global scale, and it is crucial that these measures are implemented in a way that supports food production and trade. The use of risk analysis coupled with sound understanding of underlying system dynamics will contribute to resilient and enduring food systems.

## Highlights

• Free trade of food, needed for food security, includes risk of disease spread.
• Science-based trade decisions protect human, animal, and plant health.
• Risk analysis capacity and use increases local and global food system resilience.
• Movement bans can counterproductively increase disease spread.
• Accounting for system complexity encourages sustainable and effective policy.

## 1 Introduction

Food security has been defined as “a situation that exists when all people, at all times, have physical, social and economic access to sufficient, safe and nutritious food that meets their dietary needs and food preferences for an active and healthy life” (Barrett, 2010). Despite reductions in hunger and poverty levels, over 26% of the world population still experiences moderate or severe food insecurity, with the majority in Asia and Sub-Saharan Africa (FAO, IFAD, UNICEF, WFP and WHO, 2019). Food insecurity is an important item in the international agenda; the second goal of the 2030 Agenda for Sustainable Development, adopted by all United Nations (UN) Member States in 2015, is to “end hunger, achieve food security and improved nutrition and promote sustainable agriculture” (United Nations, 2015). That goal is challenged by major events such as the COVID-19 pandemic, which the UN has estimated could almost double the number of people suffering from acute hunger by the end of 2020 (United Nations World Food Program, 2020). The conundrum is that although livestock production and global trade are necessary components for achieving food security, they are each vulnerable to the impacts of, as well as potential contributors to, such infectious disease events.

Animal-source foods do and will continue to play an important role in meeting energy and nutrition needs. Regions severely affected by food insecurity are experiencing both population and income growth. The total population of the 47 least developed countries is growing 2.5 times faster than that of the rest of the world. Per capita consumption of animal-source foods is increasing, driven by higher incomes and associated dietary preferences (Godfray et al., 2018; Rohr et al., 2019). Total meat consumption is projected to rise substantially through 2050, mostly in low- and middle-income countries (Godfray et al., 2018). The global livestock herd will expand accordingly. Models from the Food and Agriculture Organization of the UN (FAO) project the number of livestock in sub-Saharan Africa could nearly triple by 2050 compared to 2012, considering increasing population and incomes (FAO, 2018).

Urban areas will absorb nearly all of global population growth, underscoring the importance of adept food distribution systems within and between countries (United Nations Department of Economic and Social Affairs Population Division, 2019; Reardon et al., 2020). Most countries are more dependent on imports than 20 years ago (“The world’s food system has so far weathered the challenge of covid-19,” 2020), and an estimated 50% of people could rely on imported food by 2050 when factoring in population growth and climate change (Fader et al., 2013). International trade will play a strategic role in supporting sustainable food production and supply (FAO, 2018; HLPE, 2016). This scenario presents an opportunity for countries to participate in agricultural trade, contributing to the supply of available nutrition in rapidly growing regions while also developing their own economies (The World Bank, 2012; Parshotam, 2018). Certain regions have made progress towards that end; most notably, a number of countries in Latin America have grown into exporting agricultural economies over the last decades (OECD/FAO, 2019). Africa is on the cusp of a free trade area intended to promote intra-continental cooperation (Muchanga, 2019). The critical role of food supply chains is becoming clear during the COVID-19 pandemic (Reardon et al., 2020). Twenty-eight countries representing 67% of agricultural exports and 60% of imports have committed to maintain fair and predictable standards in order to mitigate the impact of COVID-19 on agricultural trade and food security and avoid the price spikes, volatility, and food shortages associated with restrictive and retaliatory measures (Responding to theD-1, 2020; Bouët and Laborde Debucquet, 2012).

For any combination of livestock expansion, intensification, and globalization that is necessary for food security and nutrition, there is a correlated set of public health risks (Mehrabi et al., 2020). Improved nutrition from consumption of animal products can improve health at both an individual and population level (Rohr et al., 2019), but there is also an increment on the risk for the emergence and reemergence of zoonotic and foodborne disease (HLPE, 2016; Daszak, 2007). Around 60% of all human diseases are zoonotic (Taylor et al., 2001), and nearly 50% of zoonotic diseases that emerged in humans since 1940 were associated with agricultural drivers (Rohr et al., 2019). Furthermore, a highly connected world, including the movement of people and goods, facilitates the rapid global spread of pathogens (Fèvre et al., 2006). The severe acute respiratory syndrome coronavirus 2 (SARS-CoV-2), the causative agent of COVID-19, emerged in China in December 2019 and soon reached pandemic proportions. In 2013, two other coronaviruses responsible for highly infectious swine disease (porcine epidemic diarrhea virus (PEDv) and porcine deltacoronavirus) caused a devastating epidemic in North America. PEDv is credited for the death of 10% of US domestic pigs and losses upwards of $1 billion in one year (Hou and Wang, 2019). The viruses likely spread through the movement of feed (Niederwerder and Hesse, 2018). Recent food supply disruptions associated with the global spread of pathogens are not limited to coronaviruses or swine. African Swine Fever (ASF) has since 2007 spread through Asia and Europe and resulted in major losses to the global domestic pig population: China reported a 40% decrease in pig inventory and near doubling of pork prices during the first year of their epidemic (Haley and Gale, 2020). Likewise, highly pathogenic avian influenza (HPAI) and infectious salmon anemia (ISA) are contagious viral diseases that traveled rapidly and wreaked havoc on farmers and countries that produce poultry and salmon, respectively (Barr, 2017; Mardones et al., 2013). Epidemics of COVID-19, PED, ASF, HPAI, and ISA illustrate the extent to which systems that produce and distribute food are, at the same time, potential sources and vulnerable targets of emerging disease events.

Considering the complex dynamics between food systems and environmental, economic, and social phenomena, there will inevitably be future events that shake local and global food supply chains. A review published in 2007 stated that a large reservoir of SARS CoV-like viruses in combination with cultural practices in southern China was “a time bomb”, suggesting the possible reemergence of SARS and other novel viruses (Cheng et al., 2007). Though the potential threat was identified, the precise event of COVID-19 was neither predicted nor prevented. In this sense, many epidemics may be considered black swan (low probability, high impact) events (Taleb, 2007; Paté-Cornell, 2012).

Acknowledging that such events will occur but without knowing precisely what, when, or where (Plowright et al., 2017), we emphasize the importance of well-equipped food and public health systems (Paté-Cornell, 2012). A connected and resilient food system, which is crucial to support the nutrition and health needs of a thriving global population, depends on the ability of all countries to detect and respond to such events. The capacity for risk analysis is a lever to promote participation in global markets, improve domestic animal health and food safety, and develop institutional capacity to respond to anticipated but unknown future developments. It follows that a response to the COVID-19 pandemic should be to strengthen the capacity for risk analysis within official veterinary services globally. This is a potentially high-reward lever to affect both food security and global health: by mitigating the impact of future infectious disease outbreaks (through rapid, local, effective response); by promoting each country's capacity to participate in the international trade of animal products while reducing the opportunity for disease transmission through that activity; and by reducing the probability of future events as structural and systemic food production and animal disease policies will be built on sound understanding and appreciation of local risks and tradeoffs.

In the first section of this article, we review the World Trade Organization's (WTO) provisions to promote free trade while protecting public and animal health. The second section describes the role of risk analysis in international trade and in building resilient food systems. The third section addresses whether trade bans would more effectively lower risks associated with movement of goods and animals, concluding that more restrictions may result in greater risk for pathogen introduction. In the final section, we highlight the need for integrated understanding of these complex systems to support both research and policy for resilience.

## 2 Trade, public health risks, and the SPS Agreement

The Agreement on the Application of Sanitary and Phytosanitary Measures (SPS Agreement) is one of the foundational pillars of the WTO (World Trade Organization, 2010). The SPS Agreement operates on the idea of “appropriate level of sanitary protection”, which is the level of protection that each member country determines is appropriate—or, conversely, the maximum level of risk that a country is willing to tolerate. Governments have the right to take trade-related measures to protect human, animal, and plant health, as long as they can demonstrate that the measures are based on science, are necessary, do not discriminate, and are not more restrictive than regulations applied within their own country. These measures take many forms including mandatory screening or processing protocols, or only accepting products from disease-free regions.

Five provisions in the SPS Agreement lay the groundwork for fair and consistent application of such measures. Harmonization and equivalence urge member countries to follow standards set by the relevant international bodies and to objectively recognize the value of other countries' measures when they differ. The World Organization for Animal Health (OIE), Codex Alimentarius, and International Plant Protection Convention are the recognized standard-setting bodies for animal health, food safety, and plant health, respectively. Transparency stipulates that countries provide sufficient and timely information regarding sanitary situations. Under regionalization, sanitary measures are to be relevant to a product's zone of origin, accounting for geographical and epidemiologic factors. Finally, risk analysis establishes that restrictive measures must be based on an assessment of risks, using systematic techniques and considering available scientific evidence. Through the WTO dispute settlement process, one country may challenge a restrictive measure imposed by another on the grounds of violating these terms.

In reality, these provisions are not as flawless in practice as on paper. WTO members differ in interpretation and implementation of the rules. Countries and individuals at times attempt to navigate the system to advance their own political and economic advantage (Conconi et al., 2017; Worsnop, 2017). The SPS Agreement was established in part because of widespread barriers maintained in the name of risk protection with no effective mechanism for challenging the basis of such measures. The new framework created a structure of expectation and accountability, but there is still work to be done to redistribute power and transform motivations of players involved. Even so, there is evidence that the SPS Agreement and WTO system have helped to facilitate healthy trading relationships. Of over 450 specific trade concerns raised in the SPS Committee meetings since 1995, only 14 have reached the substantive second stage of the formal process (OECD/WTO, 2019). The outcome of several disputes has been revised policies better aligned with the standards above. For example, in DS447, Argentina accused the US of, among other things, failing to adapt import regulations to reflect the epidemiologic characteristics of beef exporting regions (United States – Measures, 2015). The dispute panel found that US policies were inconsistent with the provision of regionalization, and the policy was updated to allow imports and reflect risk measures specific to the regions with and without vaccination for FMD (United States, Animal and Plant Health Inspection Service, 2015). In another example, Canada filed a complaint against the Republic of Korea for failing to align their policies with the OIE recommendations (harmonization) after the OIE had classified Canada as a country with “controlled risk” for Bovine Spongiform Encephalopathy (BSE) and defined appropriate measures under which exports could occur (Korea- Measures Affecting, 2012). Before the panel delivered a ruling, Canada and Korea reached a mutually acceptable policy revision and Korea lifted its BSE prohibition on the import of Canadian beef. One may subsequently argue that although the current system is not perfect, it represents progress compared to its predecessors.

A remaining challenge is that market access is unevenly distributed, as less developed economies are typically unable to achieve the level of risk that is deemed “acceptable” by wealthier regions. For example, only four of the 47 UN-listed least developed countries have been designated disease-free for trade purposes by the OIE for any of the eligible animal diseases (World Organization for Animal Health (Office International des Epizooties), 2020; The Least Developed Country Category, 2018 Country Snapshots, 2018). Food safety is challenged by fragmented food systems and poor capacity to enforce regulation (Grace, 2015). Some surveys for antimicrobial residues in African countries report residues detected in greater than 20% of meat and milk samples (Mensah et al., 2014). This situation sets up a challenging dynamic: countries with few resources and high disease challenges to start with are unable to access the premium markets that would provide resources and incentive to further develop their agricultural economy and health infrastructure (Unnevehr and Ronchi, 2014). In an attempt to mitigate those challenges, the Standards and Trade Development Facility (STDF) was established in 2001 to support developing markets to gain and maintain market access through compliance with sanitary and phytosanitary standards (World Trade Organization, 2010; Standards and Trade Development Facility, 2019.

## 3 Risk analysis and resilient local and global food systems

The SPS Agreement formalized the role of risk analysis in food trade policy (World Trade Organization, 2010). Risk analysis is a systematic approach for characterizing the expected outcome and impact of an event, considering the variability in the system and uncertainty in our knowledge (Kaplan and Garrick, 1981). The OIE approach consists of four interwoven steps: hazard identification (what could go wrong?), risk assessment (what is the probability and consequence if it happens?), risk management (how to mitigate that risk?), and risk communication (what should we say about it, how, to whom?) ((World Organization for Animal Health Office International des Epizooties, 2019)). An analysis may be carried out by an importing country (to guide decisions about a particular product or following a change in a region's sanitary status) or by an exporting country (to demonstrate that a product or region does not present substantial risk).

When there is not enough evidence to assess risk, the SPS Agreement allows for precautionary measures. Examples include disease outbreaks with an evolving and uncertain epidemiologic situation or the use of new technology for which the risks are unclear. Decisions based on uncertainty are inherently provisional and need updating as new information is available (Hansson, 2016; Foster, 2009). Uncertainty-based restrictions can tread the line between precaution and opportunism – e.g., bans on North American swine exports during the 2009 H1N1 outbreak, following the unfortunate designation of the disease in the media as “swine flu” and despite WHO and OIE recommendations that deemed such measures unnecessary (Worsnop, 2017). Further friction arises from differences in interpretation (how much uncertainty is enough to exercise precaution?). Trade disputes involving these issues have stalled in various stages of negotiation (World Trade Organization, 2020a, 2020b) and have generated debate around what it means to exercise science-based precaution and the role of public opinion (Epps, 2008; Goldstein and Carruth, 2004). In several cases these disputes have reflected or escalated highly politicized interests, such as bans by the European Union regarding beef raised with hormones, poultry processing methods, and biotechnology (Orden and Roberts, 2007; Johnson, 2014, 2015).

The ability to execute and communicate risk analyses is therefore vital for countries to participate and self-advocate in international trade while minimizing their exposure to hazards. Furthermore, the capacity for risk analysis strengthens within-country public health systems in their prevention, detection, and response to threats (Bastiaensen et al., 2017; Hoffmann, 2010a). Equipping each country and region in this way will strengthen trade relationships, local food and public health systems, and global resilience in the face of a future pandemic or shock to the food system.

The OIE specifies the application of risk analysis as a required competency for members of a country's veterinary authority ((World Organization for Animal Health Office International des Epizooties, 2012)). Official veterinary services manage animal health and food safety, carry out surveillance activities, and inform risk-based policy and planning (Zepeda et al., 2005). A review of OIE-conducted evaluations of the Performance of Veterinary Services (PVS) in 44 African countries found that all except three countries lacked the technical capability to conduct risk assessments in compliance with OIE standards (Bastiaensen et al., 2017), and the report suggests that developing and in-transition countries globally lack risk analysis capacity. Building such capacity can encourage a virtuous cycle that begins with using information available, albeit unrepresentative data or expert opinion, to identify and prioritize areas of focus to both mitigate and better understand sources of risk. That process facilitates the concentration of resources, including opportunities such as the STDF, to expand and strengthen infrastructure for data collection, diagnostic testing, and communication among stakeholders. Once a country has developed the capacity to minimize, monitor, and mitigate known risks in food systems, then they are also equipped to detect and respond to the unexpected.

Epidemics are black swan (low probability, high impact) events that cannot be precisely predicted and therefore prevented. Rather, the ability to weather such events depends on a resilient system (Cox, 2020). In this context, resilience includes the readiness to anticipate and mitigate the impact of epidemic events that are expected to happen without knowledge of when or where they will occur. A resilient global food system will be characterized by redundant layers of vigilance and all regions outfitted to respond to and mitigate the impact of novel threats.

## 4 Counterproductive impact of trade bans on disease spread

Some argue, particularly in a crisis as painful as the COVID-19 pandemic, that it would be prudent to minimize rather than encourage the movement of animals and products between regions (Goodhart, 2020; Legrain, 2020). However, restrictive measures such as trade and movement bans may lead to more underground, and therefore unregulated, movement of goods. For example, public health authorities encouraged the closure of live bird markets during the H7N9 epidemic in China in 2013–14, attempting to limit human cases. That decision prompted altered and unauthorized poultry trading that spread the virus to previously uninfected areas (Li et al., 2018). A given volume of activity will pose a greater risk of transporting and introducing biological hazards when it is unregulated. In other words, permitted and formal avenues for trade can foster a lower total likelihood of disease spread than measures that divert the movement of goods into underground channels (Marcos and Perez, 2019; Kwan et al., 2017).

The illegal movement of animals and animal products has an established role in the spread of infectious disease (Fèvre et al., 2006; van den Berg, 2009). There are intrinsically few data on the volume of smuggled goods, but regular impound events imply extensive activity. The Animal and Plant Health Inspection Service of the US Department of Agriculture reports that 8000 pork products are confiscated annually (Jurado et al., 2019). In spring 2019, a 1 million-pound shipment of illegal pork from China was confiscated in Newark, NJ (during the period that the ASF epidemic was decimating the Chinese swine industry) (Nieto-Munoz, 2019). Several countries, upon testing seized pork products, have found them to be carriers of ASF (Jurado et al., 2019), and there are several past ASF outbreaks attributed to illegally imported goods (Costard et al., 2013). Foot and mouth disease and classical swine fever outbreaks have also been linked to illegal entry of meat or meat products (Costard et al., 2013). Unregulated animal movements can have direct public health impacts as well. The high prevalence of human brucellosis in Saudi Arabia has been attributed to unregulated livestock imports from Africa (Fèvre et al., 2006); 21 people in France required post-exposure prophylaxis for rabies after contact with an illegally imported rabid dog (Fèvre et al., 2006).

## 5 Integrated understanding for effective applications

The goal of risk analysis is to characterize variable and uncertain events in order to plan and implement strategies for a desirable outcome (Kaplan and Garrick, 1981). Those events take place in the context of dynamic, complex systems. Failing to understand or account for that complexity can lead to policy resistance (the tendency for interventions to be defeated by the response of the system to the intervention itself) and unintended consequences (Sterman, 2000). Conversely, the drivers of system behavior comprise a palette of policy levers for risk management that include social, cultural, and economic incentives (Rich and Perry, 2011). Models by design require simplification, but awareness of simplifying assumptions helps users to appropriately interpret and contextualize model output (Brisson and Edmunds, 2006). We want to draw attention to two types of assumptions and their implications for designing and interpreting risk analyses.

First, risk is a function of human choices and behaviors (Perrings et al., 2018; Hoffmann, 2010b). In livestock systems, such behaviors include decisions to implement biosecurity measures, report a notifiable disease when suspected, and use pharmaceuticals as regulated. These actions result from socioeconomic factors that may be laborious to include in risk assessment but nonetheless should be explored. Insights can be used to identify behaviors that can be modified, and incentives that may be implemented, to mitigate public health risks (Rich and Perry, 2011; Wolf, 2017; Perrings et al., 2014).

Second, relationships between variables may be non-linear and/or bi-directional (e.g. there may be threshold effects or feedback loops). Models that fail to capture these dynamics may inaccurately estimate baseline risk, risk reduction, or the value of costs and benefits associated with proposed measures (Williams and Thompson, 2004; Brisson and Edmunds, 2003). In livestock value chains, such relationships include those between incentive structures, foodborne disease, and producer and consumer actions (Williams and Thompson, 2004; Rich, 2007).

Integrated and simulation models can incorporate behavioral drivers and dynamic complexity directly into assessments of risk (Hayashi et al., 2019; Ford et al., 2019; Rich et al., 2013; Duintjer Tebbens et al., 2015). Complex quantitative models are not always practical or appropriate. That said, mental models (on the part of modelers and decision makers) that look for the complexity in the system and wonder about its impact on an assessment's output will produce more productive discussion and effective policy conclusions than those which blindly assume simplicity (Sterman, 2001).

Such mental models – our understanding of the system we are navigating – can be informed by field research and formal modeling studies that elucidate livestock food systems. Food security, infectious disease, ecosystem services, economic development, and the movement of people and animals are all linked through the type of dynamic complexity described above (Rohr et al., 2019; Grace et al., 2017; Carter and Barrett, 2006). By studying these dynamic relationships as an integrated and tangled whole rather than individual silos (Ingram, 2011; Restif et al., 2012), we can learn to better recognize self-reinforcing behaviors and underlying mechanisms (that contribute to policy resistance and frustrated outcomes) as well as levers for effective and sustainable change.

## 6 Conclusion

Food production and distribution systems, including open and global trade, are critical for meeting the twin goals of food security and global health in the 21^st^ century. In this highly connected and evolving world, unpredictable yet impactful events will continue to emerge. The COVID-19 pandemic has highlighted the need to detect, report, and implement effective response measures globally: in importing and exporting countries and at the origin and destination of potential epidemics. The capacity and use of risk analysis coupled with sound understanding of underlying system dynamics will contribute to resilient and enduring food systems. By being both proactive and reactive at local and global scales, the world should progress toward the ultimate objective of promoting societal growth and food security by handling risk with awareness rather than fear, and with innovation and solidarity rather than isolation.

## Declaration of Competing interest

The authors declare that they have no known competing financial interests or personal relationships that could have appeared to influence the work reported in this paper.
